# HIV-1 Nef promotes the localization of Gag to the cell membrane and facilitates viral cell-to-cell transfer

**DOI:** 10.1186/1742-4690-10-80

**Published:** 2013-07-30

**Authors:** Marine Malbec, Marion Sourisseau, Florence Guivel-Benhassine, Françoise Porrot, Fabien Blanchet, Olivier Schwartz, Nicoletta Casartelli

**Affiliations:** 1 Département de Virologie, Institut Pasteur, Unité Virus et Immunité, 28 rue du Docteur Roux, Paris F-75015, France; 2 CNRS, UMR3569, 28 rue du Docteur Roux, Paris F-75015, France; 3 Université Paris Diderot, Sorbonne Paris Cité, Cellule Pasteur, 28 rue du Docteur Roux, Paris F-75015, France

## Abstract

**Background:**

Newly synthesized HIV-1 particles assemble at the plasma membrane of infected cells, before being released as free virions or being transferred through direct cell-to-cell contacts to neighboring cells. Localization of HIV-1 Gag precursor at the cell membrane is necessary and sufficient to trigger viral assembly, whereas the GagPol precursor is additionally required to generate a fully matured virion. HIV-1 Nef is an accessory protein that optimizes viral replication through partly defined mechanisms. Whether Nef modulates Gag and/or GagPol localization and assembly at the membrane and facilitates viral cell-to-cell transfer has not been extensively characterized so far.

**Results:**

We report that Nef increases the total amount of Gag proteins present in infected cells, and promotes Gag localization at the cell membrane. Moreover, the processing of p55 into p24 is improved in the presence of Nef. We also examined the effect of Nef during HIV-1 cell-to-cell transfer. We show that without Nef, viral transfer through direct contacts between infected cells and target cells is impaired. With a *nef*-deleted virus, the number of HIV-1 positive target cells after a short 2h co-culture is reduced, and viral material transferred to uninfected cells is less matured. At later time points, this defect is associated with a reduction in the productive infection of new target cells.

**Conclusions:**

Our results highlight a previously unappreciated role of Nef during the viral replication cycle. Nef promotes HIV-1 Gag membrane localization and processing, and facilitates viral cell-to-cell transfer.

## Background

Human Immunodeficiency virus type-1 (HIV-1) contains three structural proteins: Pr55Gag (also termed Gag or p55), Pr160GagPol (GagPol) and the envelope (Env) protein. The p55 precursor is the building block of HIV-1 assembly and is necessary and sufficient for the production of viral like particles (VLPs). Gag is organized into four major domains: matrix (MA or p17), capsid (CA or p24), nucleocapsid (NC) and p6. During translation of the Gag mRNA, a ribosomal frame shift occurs at an efficiency of 5-10% and generates the 160kDa precursor GagPol fusion protein. The Pol region contains virus specific enzymes, protease (PR), reverse transcriptase (RT) and integrase (IN). Following Gag and GagPol translation, both proteins are relocated to the cell membranes and co-assemble into virus particles at a ratio of 10–20:1 [1]. Gag directs particles assembly, whereas GagPol is incorporated into viral particles following its interaction with Gag [2-4]. GagPol incorporation is crucial for infectivity since virion maturation requires the PR activity that is auto-catalytically activated during or immediately after viral budding. Of note we refer throughout the text to “Gag proteins” to indicate all Gag species (immature and mature) present in infected cells. Some processed forms of Gag can be found in the cytoplasm of infected cells but these do not seem to contribute to virus particle formation [5]. Additionally, premature Gag processing reduces the infectivity of the virions [6,7]. The site of viral assembly varies depending on the type of the producer cell (for recent reviews see [8,9] and [10]). In some cell lines (293T and HeLa), as well as in primary CD4+ T cells, assembly takes place mostly at the plasma membrane (PM) [10-12]. Assembly and budding of HIV-1 at the PM leads to both cell-free virus spreading and viral cell-to-cell transmission to neighboring cells [13,14]. In macrophages, virus assembly and accumulation have been visualized in intracellular compartments that may be connected to the extracellular milieu [15,16].

In the cytoplasm, Gag is mostly found as monomers and dimers whereas higher ordered multimers are detected once Gag has reached the membranes [17]. The binding and accumulation of Gag to the membranes is a cooperative process regulated in part by the total amount of Gag in infected cells [18].

Early studies have demonstrated the importance of Nef for efficient viral replication and pathogenesis *in vivo*: *nef*-deleted SIVmac239 displays attenuated viral replication and pathogenicity in rhesus macaques [19]. Mutations and deletions of HIV-1 *nef* have been found in virus isolates from several HIV-1 long-term non-progressors [20-22]. *In vitro*, Nef is a multi-functional protein responsible for: (1) down-regulation of cell surface molecules such as CD4, major histocompatibility complex class I and class II, CD28, and CD3, (2) enhancement of virion infectivity and stimulation of viral replication, and (3) modulation of T cell activation state (for recent reviews see [23] and [24]).

Viral particles can infect target cells both as cell-free virions and through cell-to-cell contacts. This latter mode of infection may have an important role *in vivo*, due to the tight packing of immune cells in lymph nodes [25], which represent a major site of viral replication. Cell-to-cell HIV-1 spread is up to 1000 times more efficient than infection via cell-free virus [26,27], and leads to simultaneous transmission of HIV-1 to multiple target cells [28]. The high multiplicity of infection associated with cell-to-cell transmission may also facilitate escape from host innate antiviral pressure, from some neutralizing antibodies, and from antiretroviral treatment [29-32][33,34]. *In vitro*, various modes of HIV-1 cell-to-cell spread have been described, including transfer through virological synapses (VS) and long distance interactions mediated by filopodia and nanotubes [35-37]. HIV-1 cell-to-cell spread can be divided into different steps. The first is the formation of a conjugate between one infected donor cell and one or more uninfected targets. This may lead to the second step, the formation of the VS [38]. VS are defined by the polarization of cellular and viral proteins at the site of contact between donor and target cells. HIV-1 Env proteins expressed on the surface of infected donor cells and CD4 and co-receptors on the targets stabilize cell-cell contacts, which are strengthened by cellular adhesion molecules [26,39], facilitating the transfer of newly formed viral particles to targets. Finally, viral fusion, at the cell surface or following endocytosis [40,41], will lead to productive infection, that we term here HIV-1 transmission.

Here, we describe the impact of Nef on the expression, localization, and maturation of Gag proteins in infected cells, as well as its effect on viral release and cell-to-cell transfer.

## Results

### Nef increases the amount of HIV-1 Gag proteins in HeLa cells

**Figure 1:**
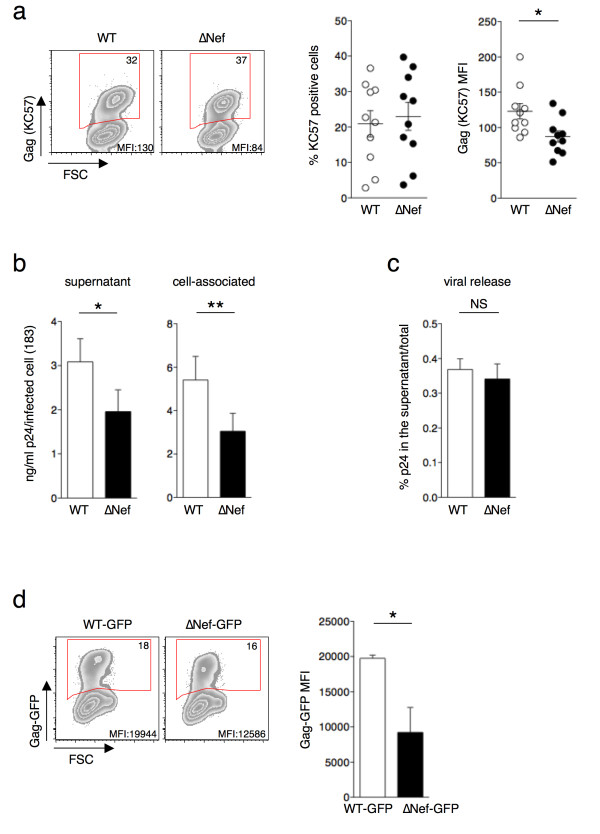
**Nef increases the amount of Gag proteins in HeLa cells.** HeLa cells were infected with VSV-G-pseudotyped wild type (WT) or *nef*-deleted (∆Nef) viruses. At day 2 post-infection cells were stained with the anti HIV-1 p24 KC57 antibody and the percentage of KC57 positive cells and their relative mean fluorescence intensity (MFI) were analyzed by flow cytometry. **(a)** Representative dot plots of infected HeLa cells (left panels). Percentage of KC57 positive cells is indicated in the top right corner of the gated population. MFI for the gated population is also indicated. A compilation of 10 independent experiments (Mean+SEM) of the percentage of KC57 positive cells and the Gag (KC57) MFI are shown (right panels). **(b)**. Levels of HIV-1 p24 (in ng/ml) in the supernatants and cell lysates derived from cells described in **(a)** and measured by ELISA using the anti HIV-1 p24 183-H12-5C antibody. **(c)** The efficiency of viral release was calculated as the ratio between the levels of HIV-1 p24 in the supernatants and the total antigen HIV-1 p24 (supernatant + cell associated). Mean+SEM is shown. **(d)**. HeLa cells were infected with VSV-G-pseudotyped WT- or ∆Nef viruses in which the green fluorescent protein (GFP) was inserted in frame with Gag-p17 (Gag-GFP). Cells were harvested at day 2 post-infection and analyzed by flow cytometry. Of note GFP signal does not distinguish between the mature and immature forms of Gag-GFP. The Gag-GFP MFI was measured on gated Gag-GFP positive HeLa cells. Representative dot plot analysis (left panel) in which the percentage of Gag-GFP positive cells is indicated in the top right corner of the gated population together with the MFI. Mean+SEM of 3 independent experiments of Gag-GFP MFI (right panel). *p<0.05; **p<0.01 (Mann Whitney test).

We first asked if Nef affects the global amount of Gag proteins (immature and/or mature) in infected cells. We infected HeLa cells with wild-type (WT) or *nef*-deleted (∆Nef) viruses pseudotyped with VSV-G. Two days later, we analyzed Gag proteins expression by flow cytometry using the KC57 antibody that recognizes an epitope contained in the p24 domain of p55. As shown in Figure 1a, even though the fraction of Gag (KC57) positive cells was similar with both viruses, the mean fluorescence intensity (MFI) of the KC57 staining in ∆Nef-infected cells was significantly reduced. We asked if this decrease corresponded to a global reduction in the amount of Gag proteins and/or to a reduced processing of Gag. To assess the relative amounts of p55 and p24 in WT or ∆Nef infected cells we performed a western blot analysis using the monoclonal anti HIV-1 p24 25A antibody (Additional file 1b,c). There was no difference in the amount of p55 in the absence of Nef, whereas p24 levels were reduced by about 3 fold. We also performed flow cytometry analysis with another anti-HIV-1 p24 monoclonal antibody 183-H12-5C [42], hereafter indicated as 183. The MFI of 183 signal was significantly reduced in ∆Nef infected cells (Additional file 1d,e), similarly to the results obtained with KC57. We then measured the global amount of Gag proteins in infected cells (cell-associated) and released in the supernatants using an ELISA assay. We used the 183 antibody, that detects mature p24 proteins, and not the p55 precursor by ELISA (Additional file 1f,g). In agreement with flow cytometry, we observed a significant reduction of cell-associated Gag proteins in the absence of Nef (Figure 1b). WT viruses were secreted in the supernatant at 3 ng /ml of p24 per infected cell, whereas in the absence of Nef this secretion was significantly lower (Figure 1b). We calculated the efficiency of viral release by dividing the amounts of p24 in the supernatants by those in the total culture (supernatant plus cell-associated) [43,44]. We did not observe differences between WT- and ∆Nef-infected cells (Figure 1c), indicating that Nef increases the overall amount of p24 in infected cells without affecting viral release, defined as the ratio of extracellular to total (extracellular+cell-associated) p24.

We confirmed the effect of Nef on the levels of Gag proteins using the HIV-1 GagGFP molecular clone, in which the GFP protein is inserted in frame at the C-terminal of the p17-MA [45]. Following GFP levels by flow cytometry allows simultaneous detection of both immature and processed forms of Gag [45]. We infected HeLa cells with VSV-G-pseudotyped WT-GagGFP or ∆Nef-GagGFP viruses. The MFI of Gag-GFP proteins was significantly reduced in ∆Nef-infected cells (Figure 1d). Thus, by using various antibodies (KC57, 183 and 25A), and different techniques (flow cytometry, ELISA and western blot) as well as an HIV-1 Gag-GFP molecular clone, we showed that there is a significant reduction of the total amount of HIV-1 Gag proteins, and of the levels of mature p24 in ∆Nef-infected cells.

### Nef increases HIV-1 p24 levels in infected primary CD4+ T cells

**Figure 2:**
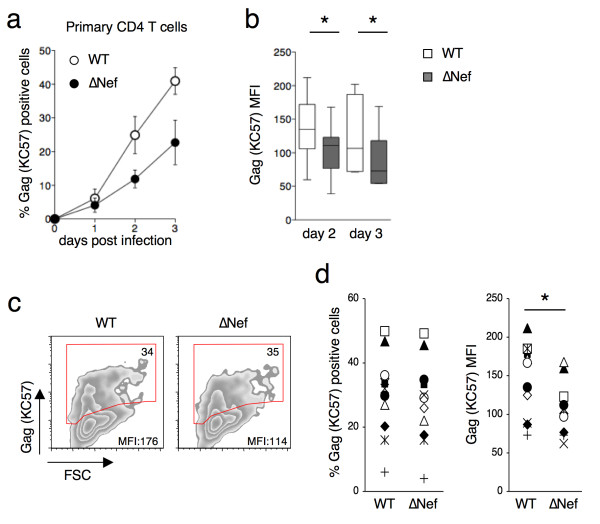
**Nef increases Gag proteins levels in infected primary CD4+ T cells.** PHA-activated primary CD4+ T cells were exposed to VSV-G-pseudotyped WT or ∆Nef (50–150 ng of p24/ml) for 3 h. The virus was washed off and the infected cells were cultured for up to 3 days. Productive infection was followed by flow cytometry of intracellular HIV-1 Gag using the KC57 anti-HIV-1 p24 monoclonal antibody. **(a)** Evolution of the fraction of Gag (KC57) positive cells at the indicated days post infection. Data are Mean±SEM of cells from three independent donors. **(b)** MFI of intracellular Gag (KC57) staining calculated on the fraction of Gag (KC57) positive cells. Maximum, Minimum and Mean of results obtained in cells described in Figure 1a are indicated. **(c)** Representative dot-plot analysis of Gag (KC57) staining of primary CD4+T cells infected with WT or ∆Nef (at day 2 post-infection). Cells were exposed to a higher viral input of ∆Nef than WT, in order to obtain similar fraction of infected cells. The percentage of Gag (KC57) positive cells is indicated in the top right corner of the gated population. MFI is also indicated. **(d)** Analysis of infected cells from 11 independent infections (8 donors), selected for the same fraction of Gag(KC57) positive cells at day 2 post-infection (left panel). The MFI of Gag (KC57) is reduced in absence of Nef (right panel). Each infection has been symbol-coded. *p<0.05 (Mann Whitney test)

We then asked whether Nef affects the levels of Gag proteins in primary CD4+T cells. We infected PHA-activated primary CD4+ T cells with VSV-G-pseudotyped WT or ∆Nef. As expected [46], VSV-G-pseudotyping rescued the infectivity of HIV-1∆*nef*, such that at 24h post-infection (p.i.) there was a similar percentage of Gag (KC57) positive cells with WT and ∆Nef (Figure 2a). However, at later time points (days 2 and 3 p.i.), when secondary rounds of replication occurred and VSV-G-pseudotyping was lost, ∆Nef spread less efficiently than WT (Figure 2a). At days 2 and 3, the MFI of ∆Nef-infected cells was significantly reduced (Figure 2b). To rule out that these differences may be due to a reduced number of ∆Nef infected cells, we used higher ∆Nef-VSV-G inoculum, in order to get the same fraction of infected cells at day 2 with WT and ∆Nef viruses. As shown in Figure 2c and 2d, when the proportion of Gag (KC57) positive cells was equivalent with both viruses, there was a significant reduction in the MFI of KC57 staining in the absence of Nef.

We performed a western blot analysis of cell lysates of primary CD4+ T cells infected with WT or ∆Nef viruses to assess the levels of p55 and p24. As shown with three independent donors (Additional file 2), there were no major differences in the amounts of p55 in WT and ∆Nef infected cells. However p24 was reduced by 25-50%, depending on the donor, in the absence of Nef. Therefore, in primary CD4+ T cells, as well as in HeLa cells, the processing of p55 into p24 is reduced in the absence of Nef.

### Nef enhances viral cell-to-cell transfer in primary CD4+ T cells

**Figure 3:**
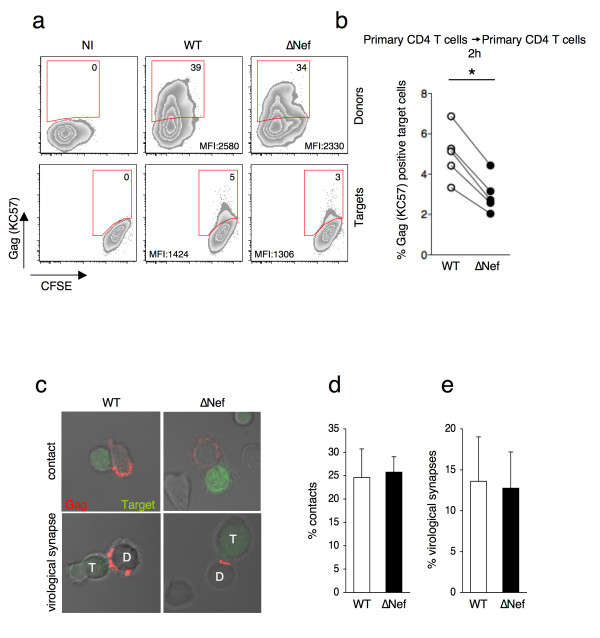
**Nef enhances viral cell-to-cell transfer in primary CD4+ T cells.** Primary CD4+ T cells were infected with VSV-G-pseudotyped WT- or ∆Nef in order to get similar levels of Gag (KC57) positive cells, or, as a negative control, left uninfected (NI). These cells were then co-cultivated with target lymphocytes pre-stained with carboxyfluorescein succinimidyl ester (CFSE) for 2h, and analyzed by flow cytometry. **(a)** Dot plot analysis of donors (upper panels) and targets (lower panels) in one representative experiment. The percentage of Gag (KC57) positive cells is indicated in the top right corner of the gated population. MFI is also indicated. **(b)** Percentages of Gag (KC57) positive primary CD4+ target cells in 5 independent experiments. **(c)** Contacts and virological synapses between infected donors (D) and uninfected CD4+ lymphocytes targets (T), visualized by immunofluorescence. Donor cells were co-cultivated with CFSE-labeled (green) target cells for 1h and stained for HIV-1 Gag proteins (red) using a polyclonal rabbit anti-Gag antiserum. A contact was defined as a tight interaction between the cells (upper panel). A virological synapse was defined as a cell conjugate in which a polarization of Gag proteins was visible at the contact zone (lower panel). **(c**, **d**, **e)**. Quantification of the percentage of conjugates **(d)** and virological synapses **(e)** formed between donor and target cells. *p<0.05 (Mann Whitney test).

We previously showed that viral replication in primary lymphocytes *in vitro* occurs mostly through cell-to-cell contacts, with very little contribution from free viral particles [27]. We investigated how WT and ∆Nef spread through cellular contacts. We infected primary CD4+ T cells for two days with VSV-G-pseudotyped WT or ∆Nef, in order to achieve the same amount of infected cells. We then used these cells as donors to transfer the infection to autologous activated CD4+ T cells. Donors were co-cultivated for two hours with target cells stained with a fluorescent dye (carboxyfluorescein succinimidyl ester or CFSE). The levels of Gag proteins were then measured by flow cytometry with the KC57 antibody. One representative staining is shown in Figure 3a and the summary of five independent experiments in Figure 3b. Following 2h of co-culture with WT-infected donor cells, we observed transfer of viral material (KC57 positive) in 3-7% of the targets. This percentage was significantly reduced when donors were infected with ∆Nef viruses. (Figure 3a and 3b).

The decreased viral transfer in the absence of Nef could be due to a reduced number of VS formed between donors and targets. We asked whether Nef might facilitate VS formation. We examined how WT and ∆Nef-infected primary CD4+ lymphocytes formed conjugates with uninfected autologous cells. Targets were stained with CFSE before being incubated with donors for 1h. Using a rabbit polyclonal anti-Gag antibody, we examined the localization of Gag proteins in cell-cell conjugates by immunofluorescence and confocal microscopy (Figure 3c). We scored approximately 100 infected cells from two different donors. The percentage of donor cells forming conjugates with targets was similar (25% of the cells) with WT and ΔNef (Figure 3d). Approximately half of these conjugates displayed a polarization of Gag proteins at the junction zone, corresponding to the VS, without significant differences between WT and ∆Nef (Figure 3e). Thus, in line with a previous report [47], Nef does not augment the capacity of infected cells to form conjugates or to polarize Gag proteins at the VS. This suggests that Nef affects the amount and/or the quality of the transferred viral material from donors to targets at a step that follows the formation of the VS.

### Nef increases viral cell-to-cell transfer in HeLa-Jurkat co-cultures

**Figure 4:**
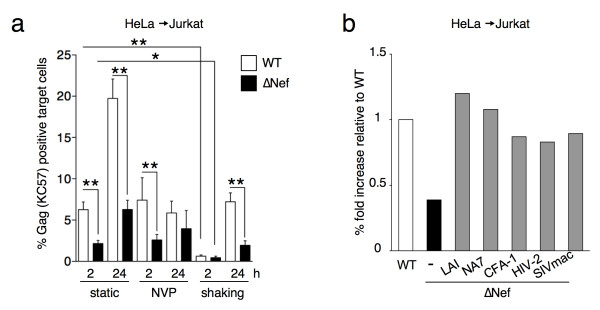
**Nef increases viral transfer from HeLa to Jurkat cells. (a)** HeLa cells infected with VSV-G-pseudotyped WT- or ∆Nef for two days and having similar levels of Gag (KC57) positive cells by flow cytometry were co-cultivated with target Jurkat cells for 2h. Jurkat cells were then harvested, and the percentage of Gag (KC57) positive cells was measured by flow-cytometry at the indicated time points. Co-cultures were performed either in static conditions, to allow cell-to-cell contacts, in the presence of reverse transcriptase inhibitor nevirapine (NVP) to evaluate productive transmission, or under gentle shaking to limit cell-to-cell contacts. A compilation of at least 4 independent experiments per condition (mean + SEM), at 2h and 24h, is depicted. **(b)**. Effect of Nef proteins from different alleles on viral cell-to-cell transfer. HeLa cells were co-transfected with HIV-1 ∆Nef and with plasmids expressing Nef from HIV-1 (LAI, NA7 and CFA-1), HIV-2 or SIVmac. Cells were then co-cultivated with Jurkat target cells, which were harvested after 2h. The percentage of Gag (KC57) positive Jurkat cells was measured at 2h and set up at 1 for WT. One representative experiment out of 2 is presented. *p<0.05; **p<0.01 (Mann Whitney test).

The absence of Nef affects viral transfer in primary CD4+ lymphocytes (Figure 3a and 3b). To gain further insights into this process, we used HeLa cells as donors and Jurkat T cells as targets. There are two main advantages of using HeLa cells as donors. The first is that viral infection does not spread beyond the first round of replication because of the absence of the CD4 receptor. The second is that targets can be easily separated at the end of the co-culture period to analyze the transferred viral material. We previously reported that this experimental system allows the analysis of cell-to-cell viral transfer and productive infection with similar results as those obtained in primary cells [43,48]. HeLa cell were infected with VSV-G-pseudotyped WT or ∆Nef for 48h and then co-cultivated with Jurkat T cells for 2h. Targets were harvested and half of the Jurkat population immediately fixed and stained to analyze viral transfer by flow cytometry using the KC57 antibody. The remaining targets were maintained in culture up to 24h to analyze productive transmission. Figure 4a represents the mean + SEM of at least four experiments. With the WT virus, around 5% of the targets were Gag (KC57) positive at the end of the 2h-coculture, and this percentage further increased to about 20% after 24h. The Gag (KC57) signal detected at 24h mostly corresponded to newly synthesized viral proteins, since it was significantly reduced when the target cells were incubated with the reverse transcriptase inhibitor nevirapine (NVP) (Figure 4a). In the absence of Nef, the fraction of positive cells was significantly reduced to 2% after 2h. The infection then progressed slower than with the WT virus, reaching about 5% at 24h.

We then verified that in this short-term co-culture system, infected cells mostly acquired the infection through direct contacts with donor cells, with a minimal contribution of free virions released in the medium. We previously reported that maintaining infected lymphocytes under gentle shaking prevents infection through cell-to-cell contacts [27,48]. Shaking the HeLa-Jurkat co-culture significantly reduced the number of Gag (KC57) positive cells at 2h (Figure 4a), confirming that in this system cell contacts are the major route of viral transfer. Interestingly, after 24h, 8% of the targets maintained in gentle shaking during the co-culture with WT-infected donor cells were Gag (KC57) positive. This residual percentage may represent the contribution of the few cell-cell contacts that could have occurred under shaking, or low levels of infection achieved by cell-free virions produced in the co-culture. Notably, ∆Nef transfer and spread were significantly reduced in shaken cultures. Of note, to describe more precisely the efficiency of recognition of p55 and p24 by KC57, we used HeLa cells transfected with WT and ∆PR HIV. In these cells, which over-express viral proteins, KC57 recognized both viruses, although the MFI of ∆PR transfected cells appeared 3–4 fold lower than WT (Additional file 3a). When these donors were co-cultivated with Jurkat cells for 2 h, some viral material was transferred. KC57 efficiently recognized the WT viral material, but not the ∆PR (Additional file 3b). However, ∆PR was transferred to Jurkat cells, as visualized by Western blot (Additional file 3c). We conclude that KC57 efficiently recognizes processed p24, and less efficiently the p55 precursor.

We then asked whether expression of Nef *in trans* in the donor cell rescues the ∆Nef defect in transfer. Co-transfection of ∆*nef* proviral DNA with a Nef-encoding plasmid (Nef-LAI) in HeLa cells enhanced ∆Nef transmission to the levels achieved with WT (Figure 4b). Two HIV-1 primary Nef alleles (from the NA7 and FA01 viral strains) [49,50] as well as HIV-2 and SIVmac Nef proteins enhanced ∆Nef transmission (Figure 4b).

Altogether, these results show that Nef increases by 2 to 3-fold the transfer of viral material to targets at 2h in the HeLa-Jurkat co-culture system. Consequently, the presence of Nef significantly enhances productive viral infection in target cells at 24h. Various Nef proteins from primary HIV-1 strains, and from HIV-2 and SIV isolates, enhanced viral cell-to-cell transfer, strongly suggesting that this function is conserved among primate lentiviruses.

### Nef increases HIV-1 p24 localization at the plasma membrane of HeLa cells

**Figure 5:**
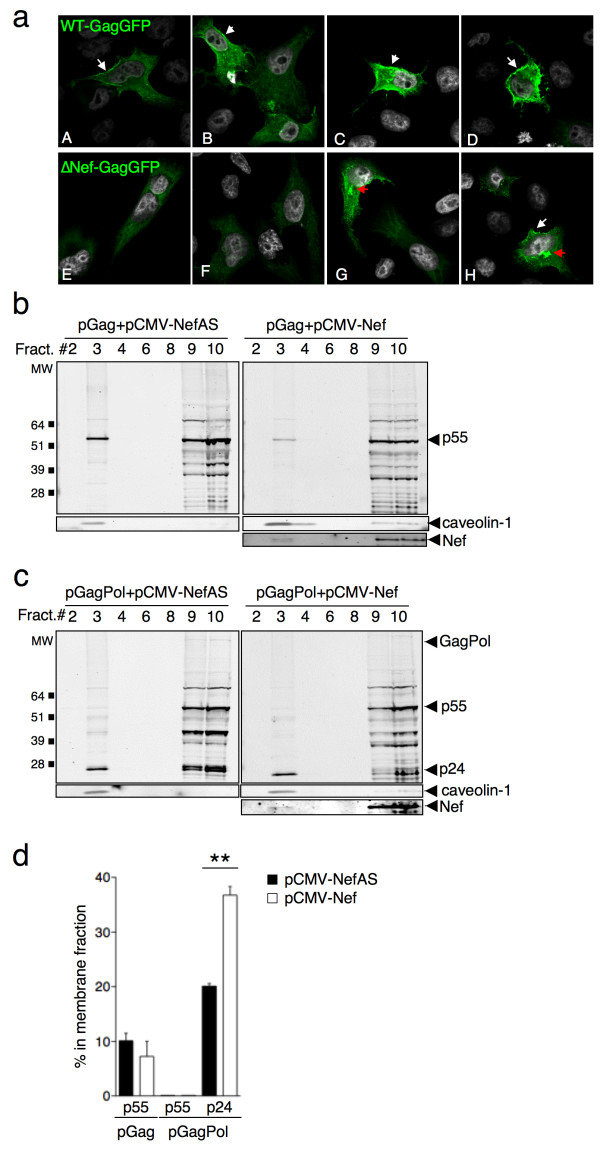
**Nef increases HIV-1 p24 localization at the plasma membrane of HeLa cells. (a)** HeLa cells were infected with VSV-G-pseudotyped WT- or ∆Nef-Gag-GFP viruses and plated on coverslips. 48h after infection cells were fixed and analyzed by confocal microscopy. Representative images for WT-GagGFP infected (A-D) and ∆Nef-GagGFP (E-H) are shown. White arrows indicate Gag-GFP proteins localized at the plasma membrane. Red arrows point to Gag-GFP proteins accumulating intracellulary. **(b**, **c)** HeLa cells were transfected with a plasmid coding for Gag (pGag) or GagPol (pGagPol) together with a plasmid coding for HIV-1Lai Nef protein (pCMV-Nef) or a control plasmid in which the Nef sequence was in antisense (pCMV-NefAS). 48h after transfection, dounce-homogenized cells were subjected to membrane flotation analysis. Panels **(b)** and **(c)** show representative western blots probed with an HIV-1 p24 monoclonal antibody (25A). Numbers on top of each lane indicate the loaded fractions. Fractions 2–4 and 8–10 correspond to membranes and cytoplasm, respectively. The immature (p55) and mature (p24) forms of Gag/GagPol are indicated. The blots were also probed with an anti-caveolin-1 antibody. **(d)** The percentages of the different Gag proteins in the membrane fractions were calculated and the mean+SEM of 3 independent experiments is shown in **p<0.01 (Mann Whitney test).

Since Nef positively contributes to HIV-1 cell-to-cell transfer, we further analyzed the effect of Nef on Gag proteins in infected donor cells. We asked if Nef could impact the intracellular localization of Gag proteins. We infected HeLa cells with VSV-G-pseudotyped WT-GagGFP or ∆Nef-GagGFP virus and visualized the localization of Gag-GFP proteins by confocal microscopy. The majority of WT-GagGFP-infected cells showed a high GFP expression (Figure 5a upper panels A-D), confirming the results obtained by flow cytometry (Figure 1d). The Gag-GFP signal was distributed in the cytoplasm and at the plasma membrane (Figure 5a, panels A-D). In the absence of Nef, the Gag-GFP signal was lower (Figures 1d and 5a, lower panels E-H), and observed mostly in the cytoplasm, with a reduced localization at the plasma membrane.

These experiments suggest that Nef affects the intracellular localization of Gag proteins in infected cells. However, this analysis does not allow discrimination between unprocessed and processed Gag, because the GFP signal is associated with both forms of the viral protein.

To further document this process, we performed subcellular fractionations and biochemical analysis of Gag-expressing HeLa cells, in the presence or absence of Nef. We used three different types of Gag-expressing cells: cells expressing only the Gag precursor, cells expressing only the GagPol precursor, and HIV-1-infected cells. To this end, we first co-transfected HeLa cells with a CMV-based plasmid coding for either Gag (pGag) or GagPol (pGagPol), along with a plasmid coding for HIV-1 Nef protein (pCMV-Nef) or a control plasmid (pCMV-NefAS, in which the *nef* sequence was cloned in antisense). Forty-eight hours following transfection, cell lysates were prepared by dounce-homogenization and a flotation assay was performed. This assay allows the separation of the different cell compartments in a 10-65-90% sucrose gradient [17]. After ultracentrifugation, ten fractions were collected, beginning with the least dense (containing the membranes), and ending with the most dense (corresponding to the cytoplasm). The proteins in each fraction were then precipitated with tricloroacetic acid (TCA), separated by SDS-page, and visualized by western blotting using the 25A anti-HIV-1 p24 monoclonal antibody. Representative western blots with Gag and GagPol are shown in Figure 5b and 5c, respectively. The fractions were also analyzed with an anti-caveolin-1 antibody. As expected, caveolin-1 was mostly localized in the membrane fractions (Figure 5b and 5c). Nef was present only in the cells transfected with the pCMV-Nef plasmid.

The relative densitometric intensities of p55 and p24 were quantified in three independent experiments (Figure 5d). When the Gag precursor was expressed alone, about 10% of p55 was localized in membranes. This distribution was not modified by Nef (Figure 5d). With the pGagPol plasmid, the main Gag species were p55 and p24 but other intermediary products were also visualized. The GagPol precursor was barely detectable, most likely because it was rapidly processed by the viral protease. p55 was detected only in the cytoplasmic fraction, irrespective of the presence of Nef (Figure 5c and 5d). HIV-1 p24, instead, was distributed between the membranes and the cytoplasm. In the absence of Nef, about 20% of the protein was localized in membranes. Interestingly, this percentage increased significantly (reaching approximately 35%) in the presence of Nef (Figure 5d). We then performed similar experiments to track the distribution of Gag proteins in HeLa cells infected with VSV-G-pseudotyped WT or ∆Nef viruses. As with GagPol-transfected cells, the accumulation of HIV-1 p24 in the membrane fractions was increased in the presence of Nef (Additional file 4).

Altogether, these experiments show that Nef promotes the accumulation of HIV-1 p24 in the membrane fraction of HIV-1-infected or GagPol-expressing HeLa cells, which may contribute to efficient cell-cell transfer.

### Nef induces HIV-1 p55 and p24 localization in membranes of infected primary CD4+T cells

**Figure 6:**
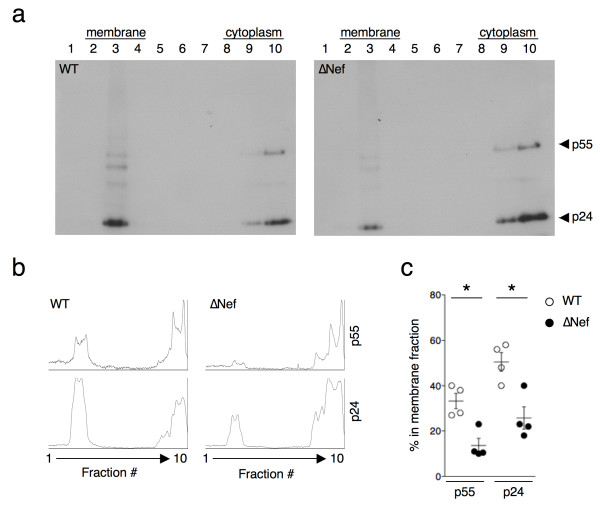
**Nef induces HIV-1 p55 and p24 localization in membranes of infected primary CD4+T cells. (a)** Primary CD4+T cells were infected with VSV-G-pseudotyped WT or ∆Nef. Two days post-infection cells were collected, dounce-homogenized and subjected to membrane flotation analysis as in Figure 5. Representative western blots of WT- (left) or ∆Nef-infected (right) primary CD4+T cells are shown. **(b)**. Quantitative densitometry analysis of the western blots for p55 and p24. The x-axis shows the pixel location in each fraction and y-axis indicates the pixel intensity. **(c)**. The percentages of p55 and p24 found in the membrane fractions was calculated and the mean+SEM of experiments performed with cells from 4 independent donors is shown. *p<0.05 (Mann Whitney test).

We examined whether Nef also modifies localization of Gag proteins in HIV-1-infected primary CD4+ lymphocytes. The cells were infected with VSV-G-pseudotyped viruses and two days later, a flotation analysis was performed on cell lysates. A representative experiment is shown in Figure 6a, and the p55 and p24 distribution in the different fractions are quantified in Figure 6b. Most of the signal corresponded to p24, and to a lesser extent to p55. In the depicted experiment, the relative membrane-associated p24 was higher with WT than with ∆Nef. This result was confirmed by compiling the mean and SEM of experiments performed on cells from four independent donors (Figure 6c). With the WT virus, 35% of Gag and 50% of p24 respectively, were localized in the membrane fractions. These percentages were significantly reduced to 15 and 25%, respectively, in the absence of Nef.

Thus, in infected primary CD4+T cells, Nef promotes the localization of p55 and p24 to the membrane fraction.

### ∆Nef-infected donor cells mostly transfer immature viral material to target cells

**Figure 7:**
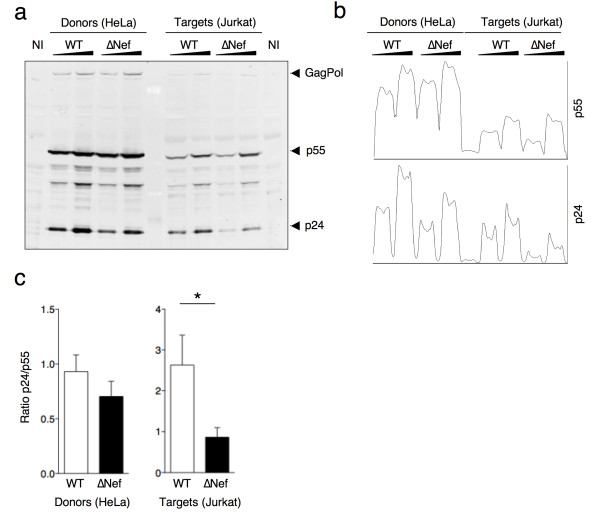
**∆Nef-infected donor cells mostly transfer immature viral material to target cells. (a)** HeLa cells infected with two doses of VSV-G-pseudotyped WT or ∆Nef viruses were used as donors to transfer the infection to Jurkat target cells as described in Figure 4. After a co-culture of 2h, donors and targets were separately harvested and cell lysates were analyzed by western blotting with a monoclonal anti-HIV-1 p24 antibody (25A). A representative experiment is shown. **(b)** Quantitative densitometry analysis of the western blots for p55 and p24. The x-axis shows the pixel location in each fraction and y-axis indicates the pixel intensity. **(c)** The ratio between mature (p24) and immature (p55) forms of Gag proteins in HeLa donors and Jurkat target cells was calculated and the mean+SEM of 3 independent experiments is shown. *p<0.05 (Mann Whitney test).

To further investigate the impact of Nef on viral transfer, we infected HeLa cells with two different doses of VSV-G-pseudotyped WT HIV-1 or HIV-1∆Nef and co-cultivated them with targets for two hours. We then performed a western blotting analysis of total Gag proteins in donor and in target cells harvested after the co-culture. A representative western blot is shown in Figure 7a with a quantification of the intensities of the p55 and p24 bands in Figure 7b. In Figure 7c, the mean and SEM of the ratio between mature and immature Gag proteins (p24/p55) are shown, as quantified in donor and target cell lysates from three independent experiments.

In donor cells, both p55 and p24 expression increased in an input dependent manner. WT-infected donor cells showed a p24/p55 ratio around 1, meaning that at the steady state, precursors and mature Gag proteins are present at similar levels. ∆Nef-infected donors had a slightly lower p24/p55 ratio, associated with reduced overall amount of p24.

We then characterized the nature of the viral material acquired by Jurkat target cells during the 2h co-culture. With WT virus, the viral material transferred was mostly mature (p24/p55 ratio of 2.5, Figure 7c). The situation was different with ∆Nef-infected donors: the amount of transferred p24 was reduced (Figure 7a), in line with the results obtained by flow cytometry (Figure 3), and the p24/p55 ratio was significantly reduced to 0.7 (Figure 7c).

Therefore, in the absence of Nef, there is not only a reduction in the amount of viral material being transferred to target cells, but also a qualitative defect in its maturation state.

## Discussion

We report here that the steady-state levels of Gag proteins in HIV-1-infected primary CD4+ lymphocytes and Hela cells are increased in the presence of Nef. Using imaging and biochemical approaches, we show that Nef changes the total amount and localization of both immature and mature forms of Gag proteins. In WT infected cells, Gag proteins are highly expressed, partially distributed in the cytoplasm and mostly localized at the plasma membrane. In the absence of Nef, expression of Gag proteins is lower, primarily cytoplasmic, and partially localized to the plasma membrane. Furthermore, the amount of HIV-1 p24 present in the supernatant of ∆Nef-infected cells is lower than that of cells infected with the WT virus (this work and [51]). However, viral release, calculated as the ratio between the extracellular levels and the total amount (released plus cell-associated) of HIV-1 p24 is unaffected. The absence of Nef in infected lymphocytes does not affect their capacity to form virological synapses, confirming previous results [47]. Once the virological synapse is formed, WT-infected cells transfer mostly mature viral particles to uninfected cells, whereas in the absence of Nef, the amount of transferred viral material is reduced and mostly immature. Together, these results strongly suggest a previously unappreciated effect of lentiviral Nef on the expression, intracellular localization and processing of Gag proteins, providing novel insights into how Nef optimizes viral replication.

How does Nef increase the amount of Gag proteins in infected cells? One possibility is that Nef, by affecting the HIV-1 long terminal repeat (LTR) activity, modulates Gag and GagPol expression. However, discrepant results have been reported regarding the effects of Nef on the transcriptional activity of the LTR [52-59] . This hypothesis will deserve further investigation. Later in the viral life cycle, Nef may prevent the degradation of Gag proteins or of other proteins involved in the trafficking or assembly of Gag and/or GagPol. In support of this, it has been reported that proteasome inhibitors partly rescue the infectivity defect of ∆Nef viruses [60]. Interestingly, Nef also increases levels of the cellular protein SOCS1 [61], a protein that is important for Gag trafficking and stability in infected cells [62].

The molecular and cellular mechanisms underlying the effects of Nef on the amount, trafficking and processing of Gag proteins remains to be further characterized. We hypothesize that Nef might positively impact the Gag biosynthetic pathway by acting at various levels. In the cytoplasm, Gag is mostly monomeric or dimeric, whereas higher ordered Gag multimers are found only at the plasma membrane [17]. Gag assembly and viral budding is a cooperative process that depends on the amount of intracellular Gag [18]. Thus, the reduced amount of Gag proteins observed in the absence of Nef could lead to an inefficient localization at the plasma membrane.

Nef is known to interact with the clathrin-dependent endocytic pathway and to modulate the surface expression of various cellular proteins [63,64]. Gag contains a dileucine-like sorting motif that regulates association with multivesicular bodies [65]. Gag interacts directly with the AP-3 complex, a component of the clathrin pathway [66,67], and trafficking of Gag to late endosomes is part of a productive particle assembly pathway prior to budding from the plasma membrane [11,66]. It is thus tempting to speculate that Nef may affect Gag trafficking through its effect on the clathrin-mediated cell sorting machinery. Nef also modulates actin dynamics by inactivating cofilin [68], while the microtubule network is dispensable for proper targeting of Gag at the plasma membrane [11]. It will be worth determining whether the effects of Nef on the actin cytoskeleton impact the overall levels of Gag proteins in infected cells.

Further work is thus warranted to determine which of these potential activities mediate the effects of Nef on Gag proteins. For instance, it may be of interest to determine which Nef mutants, known to be selectively defective in different activities of the viral protein, impact Gag localization and processing. Additionally, it has been shown that murine leukemia virus (MLV) glycosylated gag (Glycogag) proteins rescue the infectivity of Nef-defective virions [69]. It will be worth examining whether Glycogag also impacts the trafficking and processing of HIV-1 Gag.

We further report a possible consequence of the effects of Nef on Gag proteins. Using two short-term co-culture systems (co-culture of infected HeLa cells with Jurkat target cells, and co-culture of primary CD4+ T cells), we demonstrate that the quantity and quality of the viral material transferred is different in the presence or in the absence of Nef. Without Nef, the percentage of targets having received viral material is significantly reduced. Moreover, by western blot analysis, we demonstrate that the p24/p55 ratio on targets, reflecting the amount of mature viral material passing from donor to target cells is also significantly reduced in the absence of Nef. In donor cells, without Nef, we observed a slight reduction of the p24/ p55 ratio as compared to cells infected with WT virus. This raises questions about how and which viral material is actually transferred from donors to targets. Recent fluorescence microscopy methods demonstrated that viral assembly, budding and release in the supernatant are rapid processes [70,71]. It will be interesting to understand whether the dynamics of HIV-1 assembly are similar in the presence or in the absence of virological synapses [72,73] and if Nef plays a role in Gag assembly. Hubner and colleagues [63] showed that Gag proteins moving across the synapse toward the target cells originate from regions of the donor cells close to the cell-contact site. Thus, in the absence of Nef, the decreased quantity of viral material transferred to targets could be a direct consequence of the reduced amount of Gag proteins at the plasma membrane of donor cells. Moreover, Nef modulates the lipid content and the nature of the cellular proteins present at the cell membrane, a process that may enhance viral infectivity [74,75]. Since rafts and other membrane microdomains polarize at the site of viral transfer and are considered as privileged Gag assembly sites [9,76], a modulation of the composition of the cellular membranes could affect Gag/GagPol assembly and subsequent processing. At the membrane, virions bud mostly as immature particles. Maturation starts during the late phases of or immediately after budding, when the autocatalytic cleavage of the PR activates this enzyme to produce the mature viral core [77]. Nef directly binds the GagPol-p6* transframe protein, but not Gag-p6, and redirecting Nef to the endoplasmic reticulum inhibits the activity of Nef on Gag processing and virion production [78]. Gag processing occurs during Gag/GagPol assembly at the plasma membrane, but not during membrane trafficking [79] and the ratio between Gag and GagPol significantly impacts the intracellular distributions of mature Gag and the infectivity of the viral particles produced [79]. Thus Nef binding to p6* may modulate the trafficking of the viral structural proteins and affect their processing.

The increase of HIV-1 p24 in membrane fractions induced by Nef is visible in HIV-1 infected cells (HeLa or primary CD4+ lymphocytes), as well as in HeLa cells expressing only GagPol. In contrast, Nef does not seem to promote p55 accumulation at the membrane when this viral protein is expressed alone. This suggests that the increase of p55 and p24 at the plasma membrane observed in infected cells might depend on the interaction of Nef with the GagPol proteins synthesized during viral replication. Of note, a part of the p24 signal detected in the membranes fraction could also result from virus being released and re-internalized [9,11,71,80]

At later time points, this altered transfer of viral material is associated with a reduction of productive infection of target cells. This is demonstrated here after 24h of viral replication in Jurkat cells that have been separated after 2h of contact with Hela donor cells. Of note, a positive effect of Nef on viral replication was observed in primary CD4+ T cells when the sources of infection were either cell-free viruses (Figure 1a) or infected lymphocytes co-cultivated with autologous targets (not shown). The effect of Nef on Gag proteins amount and trafficking described help explain the slower kinetics of replication observed for ∆Nef viruses. Portillo and colleagues [32] showed that cell-to-cell transmission significantly increases the number of copies of viral DNA integrated in the host genome. The differences in the quantity and quality of transferred viral material between WT and ∆Nef could thus affect the number of integration events and the amount of viral proteins produced per infected cells. Moreover, there are less mature virions transferred in the absence of Nef, so the fusion events at the virological synapse could also be affected [81]. Additionally, we show that in the absence of Nef, the amount of transferred immature viral material is significantly increased. It has been recently proposed [40] that immature viral particles are first endocytosed and then undergo maturation inside the target cell. Without Nef, this “excess” of immature viral material may necessitate a longer maturation time, which may further delay viral replication.

The profile of maturation of Gag proteins in extracellular virions is considered to be similar with or without Nef ([82], and not shown). However, these analyses were generally performed on viral particles harvested after long periods of times (i.e. after a few hours to a few days). This may have masked short-term effects of Nef on the kinetics of viral maturation after extracellular release.

## Conclusion

Nef accelerates viral spread through partly characterized mechanisms. Our work describes a new role of Nef in modulating various steps of the viral Gag pathway. We demonstrate that in the presence of Nef, Gag proteins are localized more efficiently at the plasma membrane, where new virions are built. We further show that the processing of Gag into mature products is enhanced and that cell-to-cell viral transfer is more efficient. It is tempting to speculate that the activity of Nef described here is relevant to viral spread and pathogenesis *in vivo*. Nef proteins from HIV-1 primary isolates, HIV-2 and SIV strains, enhance viral cell-to-cell transfer, indicating that this function is conserved among primate lentiviruses. Infected cells are vehicles for viral spread *in vivo*[25]. In HIV-positive individuals, productively infected CD4+ T cells have a short half-life (1.6 days) [83], and consequently a short time to spread the infection. The facilitation of proper Gag proteins trafficking and processing and cell-to-cell transfer likely represent critical aspects of Nef function, which may help explain why viral loads are significantly reduced in infected individuals harboring Nef-defective viruses.

## Methods

### Cells

Jurkat (clone 20), HeLa and 293T cells were grown as described [28]. Primary CD4+ T cells were purified from human peripheral blood by density gradient centrifugation (Lymphocytes separation medium, PAA) followed by positive immunomagnetic selection (Miltenyi). About 98% of cells were CD4+CD3+. For activation, primary T cells were treated with phytohemagglutinin (PHA, 1 μg/ml) (Remel, Dartford, UK) for 24h at 37°C and then cultured in interleukin 2 (IL-2)-containing medium (50 IU/ml) for one week before being used.

### Virus, Infections and Transfections

Virus stocks were prepared by transfection of 293T cells ([28]). For some experiments viral supernatants were ultracentrifuged at 22000 rpm for 2h at 4°C through a 20% sucrose cushion. Cells were infected with the X4 HIV-1 strains NL4-3 (WT), NL4-3∆Nef (∆Nef), NL4-3-GFP (WT-GFP) or NL4-3∆Nef-GFP, pseudotyped with the vesicular stomatitis virus G protein (VSV-G) to allow more efficient viral entry. For Nef complementation experiments, HeLa cells were co-transfected with pNL4-3∆*nef* along with plasmids expressing HIV-1 Nef LAI, NA7 and FA01 primary alleles, HIV-2 or SIV Nef [49,50]. Co-transfections of Nef and Gag or GagPol plasmids were performed at a ratio of 2:1, respectively. For transfections, Lipofectamine2000 (Invitrogen) or Metafectene (Biontex Laboratories) was used following manufacturer’s instructions. Transfected cells were then co-cultivated with target Jurkat cells or used in flotation assay as described below.

### Anti Gag proteins antibodies

Anti-HIV-1 p24 monoclonal antibody KC57 (clone FH190-1-1; Coulter); anti-HIV-1 p24 monoclonal antibody produced by the hybridoma cell line 183-H12-5C (NIH AIDS Reagent Program, Division of AIDS, NIAID, NIH)); mouse monoclonal anti-HIV-1 p24 (clone 25A, Institut Pasteur).

### Flow cytometry

To measure HIV-1 Gag proteins expression, infected cells were permeabilized in PBS/1% BSA/0.01% Sodium Azide/0.5% Saponin (Sigma) and stained using different anti HIV-1 p24 antibodies: anti-HIV-1 p24 phycoerythrin mAb KC57 was diluted 1:500; anti-HIV-1 p24 monoclonal antibody 183-H12-5C was diluted 1:1000. Secondary antibody anti-mouse Alexa-647 (Invitrogen) was used to detect 183-H12-5C primary antibody. Isotype-matched mAbs were used as negative controls. Samples were analyzed with either a FACSCalibur instrument (Becton Dickinson) and CellQuest software or a BD FACSCanto II (BD Biosciences) and Diva software.

### Analysis of HIV-1 cell-to-cell transfer

HIV-1 cell-to-cell transmission assay has been previously described [27,48]. Briefly, 48h after transfection or infection, equivalently (±5% accordingly to flow cytometry staining with KC57 anti-HIV-1 p24 antibody) infected HeLa donor cells were co-cultivated with target T cells labelled with Carboxyfluorescein succinimidyl ester (CFSE-Invitrogen). Staining of target cells with CFSE (final concentration 500nM) was performed in RPMI without fetal bovine serum (FBS) for 5 minutes at 37°C. Cells were then washed once in RPMI without FBS, resuspended in complete media and co-cultivated with donors for 2h. Targets were then harvested, washed, and incubated at 37°C up to 24h. At the indicated time points, cells were stained using the anti-HIV-1 p24 KC57 antibody and analyzed by flow cytometry. When stated, the reverse-transcriptase inhibitor nevirapine (NVP, at 25 nM) was added during the co-culture and maintained during the assay. Co-cultures of primary CD4+ T cells were performed similarly, except that donor and target cells were kept together for 2h.

### Immunofluorescence

Analysis of conjugates and virological synapses has been previously described [28]. Briefly, HIV-1-infected donor cells were mixed with targets pre-labelled with CFSE (200 nM, Invitrogen) at a 1/1 ratio and loaded on polylysine-coated coverslips. After 1 h at 37°C, cells were fixed 10 min with 4% PFA. Cells were stained with a rabbit polyclonal anti-Gag proteins (a kind gift of Pierre Boulanger) [84] and analyzed by confocal microscopy on a Zeiss LSM700 microscope. HeLa cells were plated on glass coverslips and the day after transfected with proviral DNA coding for the pNL4-3 or the *nef*-deleted counterpart expressing the green fluorescent protein (GFP) in frame with the HIV p17 protein [45]. Two days after transfection, cells were fixed and analyzed by confocal microscopy on a Zeiss LSM-700 microscope.

### Membrane flotation assay (equilibrium flotation centrifugation)

The membrane flotation assay was performed as previously described [17]. In brief, approximately 1×10^7^ cells were washed three times with NTE buffer (100 mM NaCl, 10 mM Tris [pH 7.4], 1 mM EDTA) and resuspended in 500 μl of hypotonic buffer (10 mM Tris [pH 7.4], 1 mM EDTA) supplemented with protease inhibitors. Samples were lysed by dounce homogenization and adjusted to 150 mM NaCl and 1 mM MgCl2. Nuclei and intact cells were removed by centrifugation for 10 minutes at 1000× g, 4°C. Thereafter, 350 μl of supernatant was mixed with 1650 μl of 90% sucrose solution (prepared in 150 mM NaCl, 10 mM Tris [pH 7.4], 1 mM EDTA, 1 mM MgCl2) and overlayed with 6.5 ml of 65% and 2.5 ml of 10% sucrose solutions prepared similarly and supplemented with protease inhibitors. Centrifugation was performed in a Beckman SW41 Ti rotor at 35,000 rpm for 18 h. Ten fractions (1 ml each) were collected from the top of the gradient and used for protein analyses by western blotting using the monoclonal anti-HIV-1 p24 antibody 25A.

### Enzyme-linked immunosorbent assay (ELISA)

Viral production was monitored by measuring HIV-1 antigen p24 in supernatants and cell-associated by ELISA. Plates were coated using a monoclonal antibody anti-HIV-1 p24 183-H12-5C diluted 1:10000 and revealed using an anti HIV-1 polyclonal serum.

### Western blotting

Cells were lysed in PBS-1% Triton X-100 (Sigma-Aldrich) supplemented with protease inhibitors (Complete; Roche). Cell lysates were analyzed by SDS-gel electrophoresis using 4–12% Bis-Tris Criterion gels (BioRad) or 3-8% Tris-Acetate gels (Invitrogen). The following antibodies were used: mouse monoclonal anti-HIV-1 p24 clone 25A, mouse anti-Nef MATG020 (Transgene) or a rabbit HIV-1 Nef Antiserum (NIH AIDS Reagent Program, Division of AIDS, NIAID, NIH: Catalog #2949, from Dr. Ronald Swanstrom); rabbit polyclonal anti-caveolin-1 antibody (clone N-20, Santa Cruz Biotechnology); mouse monoclonal anti beta-actin (clone AC-15, Sigma Aldrich). HRP- or IRDye-coupled specie-specific secondary antibodies were used. Western Blot HRP quantification was performed using ImageJ software from NIH; fluorescent signals were detected and quantified using Image Studio (LICOR Odyssey).

### Statistical analysis

Statistical analyses (Mann-Witthney unpaired t test) were performed using GraphPad Prism software.

## Competing interests

The authors declare that they have no competing interests.

## Authors’ contributions

NC and OS conceived the study. MM, MS, FGB, FP, FB and NC performed the experiments and/or participated in the experimental design. MM, NC and OS wrote and edited the manuscript. All authors approved the final manuscript.

## Supplementary Material

Additional file 1 **Analysis of the recognition of Gag proteins by flow cytometry and western blot using various anti HIV-1 p24 antibodies.** (a) VSV-G-pseudotyped WT- or ∆Nef-infected HeLa cells were stained with the anti HIV-1 p24 KC57 antibody 48h after infection. The percentage of KC57 positive cells and relative MFI are indicated in the top right corner of the gated population and in the low right corner of the dot plot, respectively. (b) Lysates of infected cells loaded on a SDS-page polyacrilamide gel and blotted with a monoclonal anti-HIV-1 p24 antibody (25A) to visualize all Gag proteins. A representative western blot is shown corresponding to the dot plots shown on the left. NI: not infected (c) Mean+SEM of the p55 and p24-associated fluorescence in 3 independent experiments. (d) 48h after infection HeLa cells were stained with the anti HIV-1 p24 183 antibody. The percentage of 183 positive cells and the relative MFI are indicated. (e) Mean ± SEM of the Gag (183) MFI in 5 independent infections. (f-g): The 183 antibody preferentially recognize the mature HIV-1 p24. (f) HeLa cells were transfected with WT or ∆PR proviral DNA or left not transfected (NT). The amount of released HIV-1 p24 antigen was measured by ELISA 48h after transfection. The 183 antibody was used to coat the ELISA plates. Amount of HIV-1 p24 antigen measured before (gray bar) and after (white bar) ultracentrifugation of the supernatants on a sucrose gradient. (g) The ultracentrifuged particles were analyzed by western blotting using the 25A antibody. 1 ng of antigen p24 was loaded for the WT virus. For the ∆PR virus, undetectable by ELISA, was loaded the same volume of ultracentrifuged virus loaded for the WT. One representative experiment out of 2 is shown **p<0.01 (Mann Whitney test) Click here for file

Additional file 2 **The amount of processed HIV-1 p24 is reduced in cell lysates of primary CD4+T cells infected with ∆Nef viruses.** Primary CD4+T cells derived from PBMCs of healthy donors were sorted by immunomagnetic selection, activated with PHA and maintained in culture with IL-2 for one week before being infected with VSV-G-pseudotyped WT or ∆Nef viruses. At day 2 post infection, cells were harvested and part of them fixed, permeabilized and stained with the KC57 antibody. Cells were then analyzed also by western blotting using the 25A antibody as described in the additional file 1 and in the materials and methods. Quantification of the p55 and p24 bands was performed for each donor using the Odyssey-LICOR system. As shown in three independent donors, in absence of Nef the fluorescence associated with the p24 band was reduced by 25-50%, depending on the donor, whereas no major differences were observed in the amount of p55. Click here for file

Additional file 4 **Nef induces HIV-1 p24 localization in membranes of infected HeLa cells.** (a) HeLa cells were infected with VSV-G-pseudotyped WT or ∆Nef. Two days post-infection, cells were collected, dounce-homogenized and subjected to membrane flotation analysis, as described in Figure 5. The panels show representative western blots probed with the HIV-1 p24-specific monoclonal antibody 25A. Numbers on top of each lane indicate the loaded fractions. Fractions 2–4 and 8–10 correspond to membranes and cytoplasm, respectively. The immature (p55) and mature (p24) forms of Gag and GagPol proteins are indicated. (b) Quantitative densitometry analysis of the western blots for p55 and p24. The x-axis shows the pixel location in each fraction and y-axis indicates the pixel intensity. (c) The percentages of p55 and p24 found in the membrane fractions were calculated and the mean+SEM of 3 independent experiments is shown. Click here for file

Additional file 3 **The monoclonal antibody anti HIV-1 p24 KC57 preferentially recognizes mature p24 by flow cytometry.** HeLa cells were transfected with proviral DNA coding for WT, or ∆PR, which is defective for the viral protease. 48h after transfection, HeLa cells were used as donors for a 2h co-culture with Jurkat target cells. (a) Donors were analyzed by flow cytometry using the anti HIV-1 p24 monoclonal antibody KC57. This antibody recognizes both the Gag precursor and mature proteins in donor cells, which over-express the viral proteins. Note that the MFI of the ∆PR provirus is reduced. (b) Targets were analyzed by flow cytometry using the anti HIV-1 p24 KC57 antibody. In target cells the Gag (KC57) signal is visible with the WT virus, and barely detected with ∆PR. (c) Donor and target cells were also harvested separately and analyzed by western blotting using the anti-p24 monoclonal antibody 25A. In donor cells, Gag species from both WT and ∆PR were detected. As expected, ∆PR produced only the Gag precursor (p55). A similar profile of staining was obtained in target Jurkat cells. Of note, KC57, when used in the western blot experiment, also detects both p55 and p24 (not shown). One out of two experiments is shown. Click here for file
