# Supplementary figures and images for: HIV-1 Nef promotes the localization of Gag to the cell membrane and facilitates viral cell-to-cell transfer
Source: Retrovirology. 2013 Jul 30;10:80. doi: 10.1186/1742-4690-10-80 (PMC3734038; doi:10.1186/1742-4690-10-80)

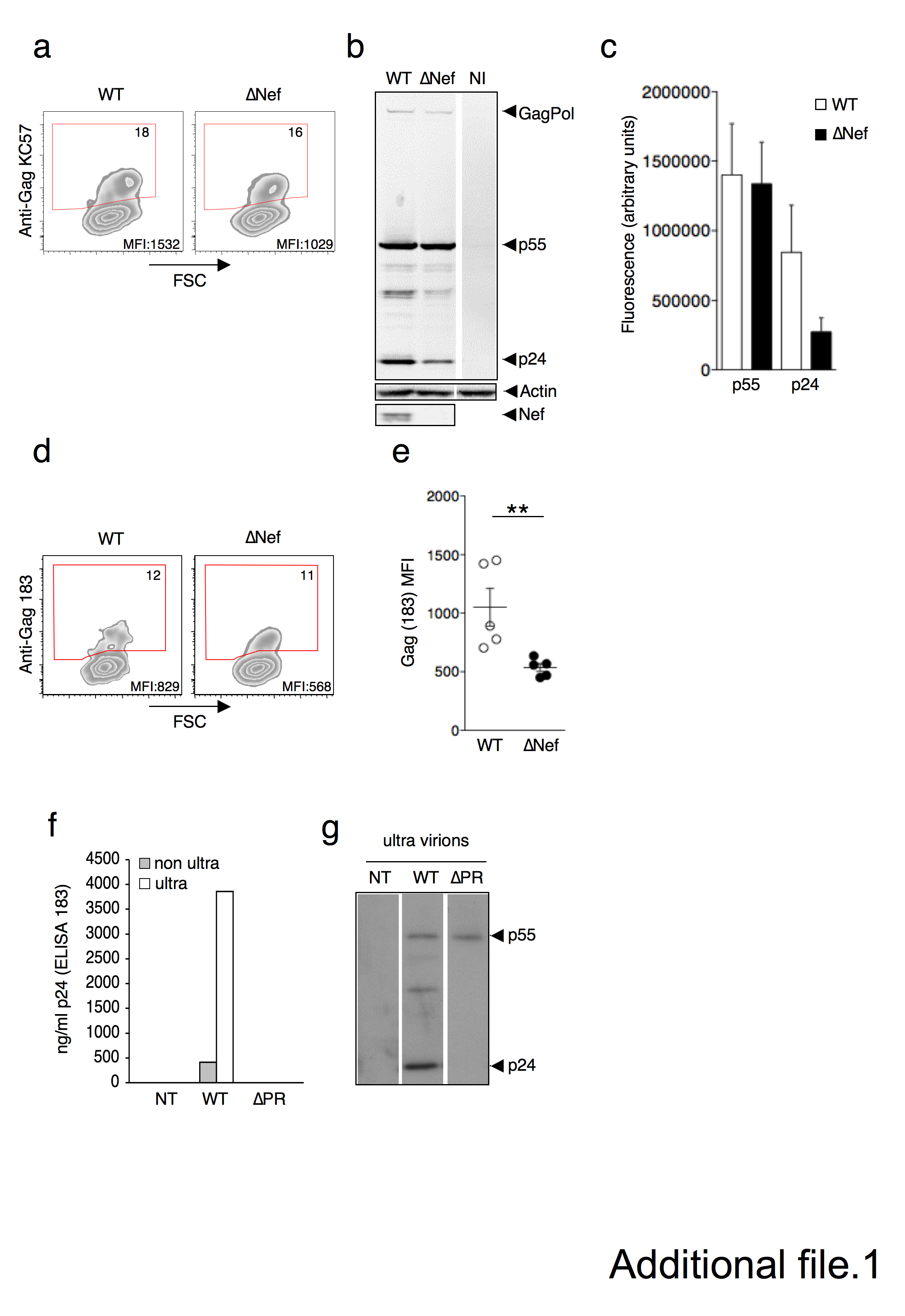

Supplement: Additional file 1 — Analysis of the recognition of Gag proteins by flow cytometry and western blot using various anti HIV-1 p24 antibodies. (a) VSV-G-pseudotyped WT- or ∆Nef-infected HeLa cells were stained with the anti HIV-1 p24 KC57 antibody 48h after infection. The percentage of KC57 positive cells and relative MFI are indicated in the top right corner of the gated population and in the low right corner of the dot plot, respectively. (b) Lysates of infected cells loaded on a SDS-page polyacrilamide gel and blotted with a monoclonal anti-HIV-1 p24 antibody (25A) to visualize all Gag proteins. A representative western blot is shown corresponding to the dot plots shown on the left. NI: not infected (c) Mean+SEM of the p55 and p24-associated fluorescence in 3 independent experiments. (d) 48h after infection HeLa cells were stained with the anti HIV-1 p24 183 antibody. The percentage of 183 positive cells and the relative MFI are indicated. (e) Mean ± SEM of the Gag (183) MFI in 5 independent infections. (f-g): The 183 antibody preferentially recognize the mature HIV-1 p24. (f) HeLa cells were transfected with WT or ∆PR proviral DNA or left not transfected (NT). The amount of released HIV-1 p24 antigen was measured by ELISA 48h after transfection. The 183 antibody was used to coat the ELISA plates. Amount of HIV-1 p24 antigen measured before (gray bar) and after (white bar) ultracentrifugation of the supernatants on a sucrose gradient. (g) The ultracentrifuged particles were analyzed by western blotting using the 25A antibody. 1 ng of antigen p24 was loaded for the WT virus. For the ∆PR virus, undetectable by ELISA, was loaded the same volume of ultracentrifuged virus loaded for the WT. One representative experiment out of 2 is shown **p<0.01 (Mann Whitney test) [file 1742-4690-10-80-S1.png]

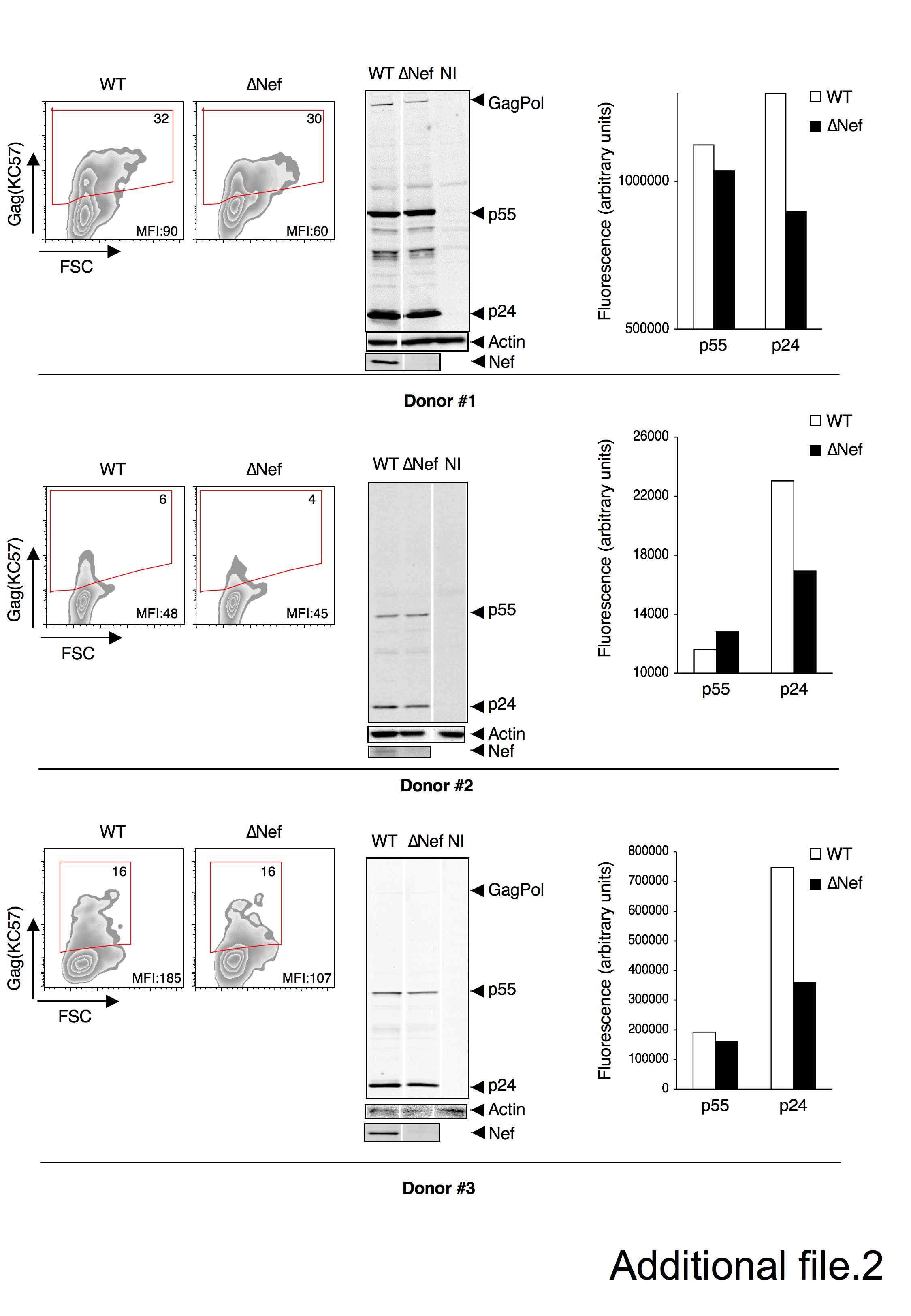

Supplement: Additional file 2 — The amount of processed HIV-1 p24 is reduced in cell lysates of primary CD4+T cells infected with ∆Nef viruses. Primary CD4+T cells derived from PBMCs of healthy donors were sorted by immunomagnetic selection, activated with PHA and maintained in culture with IL-2 for one week before being infected with VSV-G-pseudotyped WT or ∆Nef viruses. At day 2 post infection, cells were harvested and part of them fixed, permeabilized and stained with the KC57 antibody. Cells were then analyzed also by western blotting using the 25A antibody as described in the additional file 1 and in the materials and methods. Quantification of the p55 and p24 bands was performed for each donor using the Odyssey-LICOR system. As shown in three independent donors, in absence of Nef the fluorescence associated with the p24 band was reduced by 25-50%, depending on the donor, whereas no major differences were observed in the amount of p55. [file 1742-4690-10-80-S2.png]

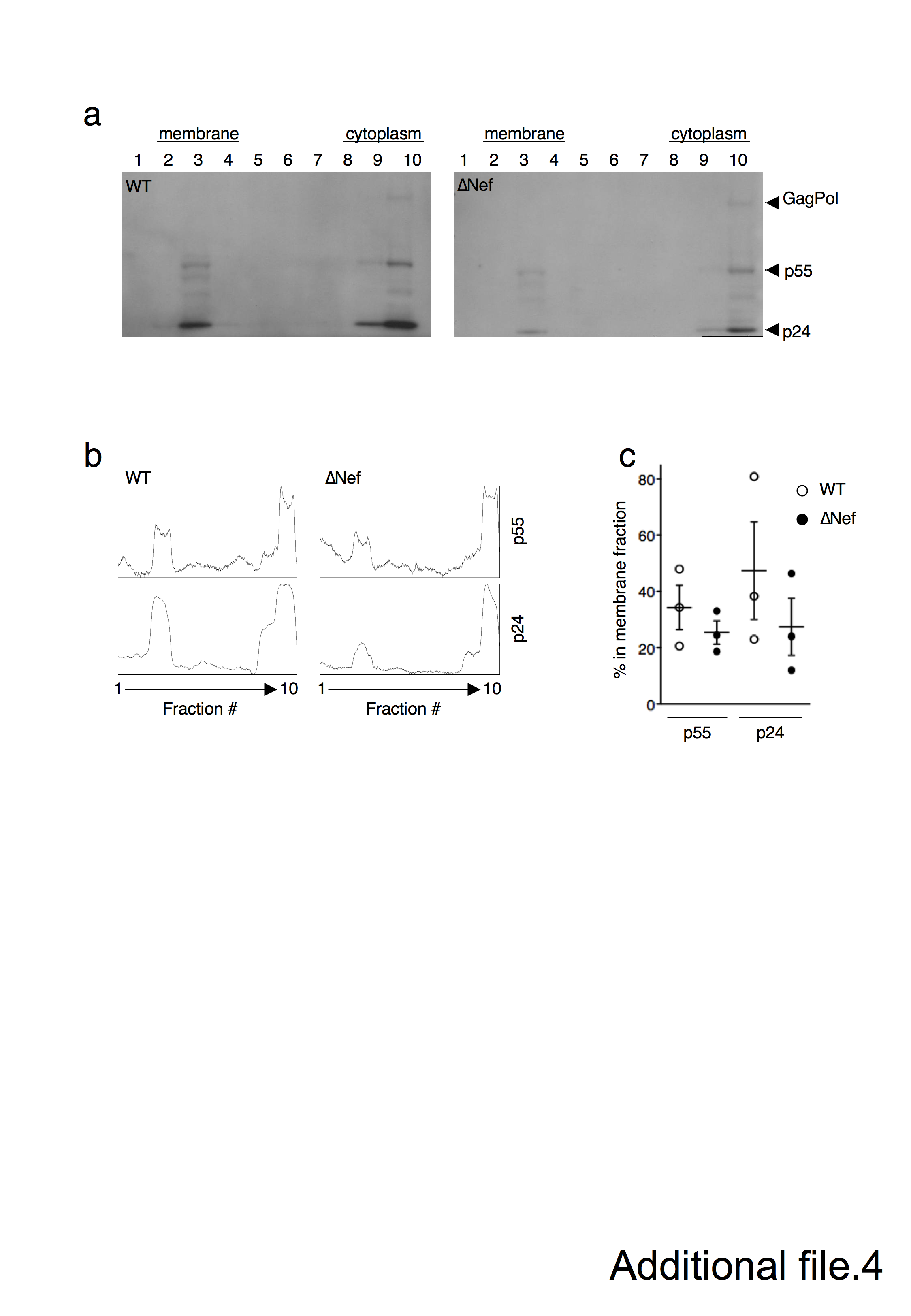

Supplement: Additional file 4 — Nef induces HIV-1 p24 localization in membranes of infected HeLa cells. (a) HeLa cells were infected with VSV-G-pseudotyped WT or ∆Nef. Two days post-infection, cells were collected, dounce-homogenized and subjected to membrane flotation analysis, as described in Figure 5. The panels show representative western blots probed with the HIV-1 p24-specific monoclonal antibody 25A. Numbers on top of each lane indicate the loaded fractions. Fractions 2–4 and 8–10 correspond to membranes and cytoplasm, respectively. The immature (p55) and mature (p24) forms of Gag and GagPol proteins are indicated. (b) Quantitative densitometry analysis of the western blots for p55 and p24. The x-axis shows the pixel location in each fraction and y-axis indicates the pixel intensity. (c) The percentages of p55 and p24 found in the membrane fractions were calculated and the mean+SEM of 3 independent experiments is shown. [file 1742-4690-10-80-S4.png]

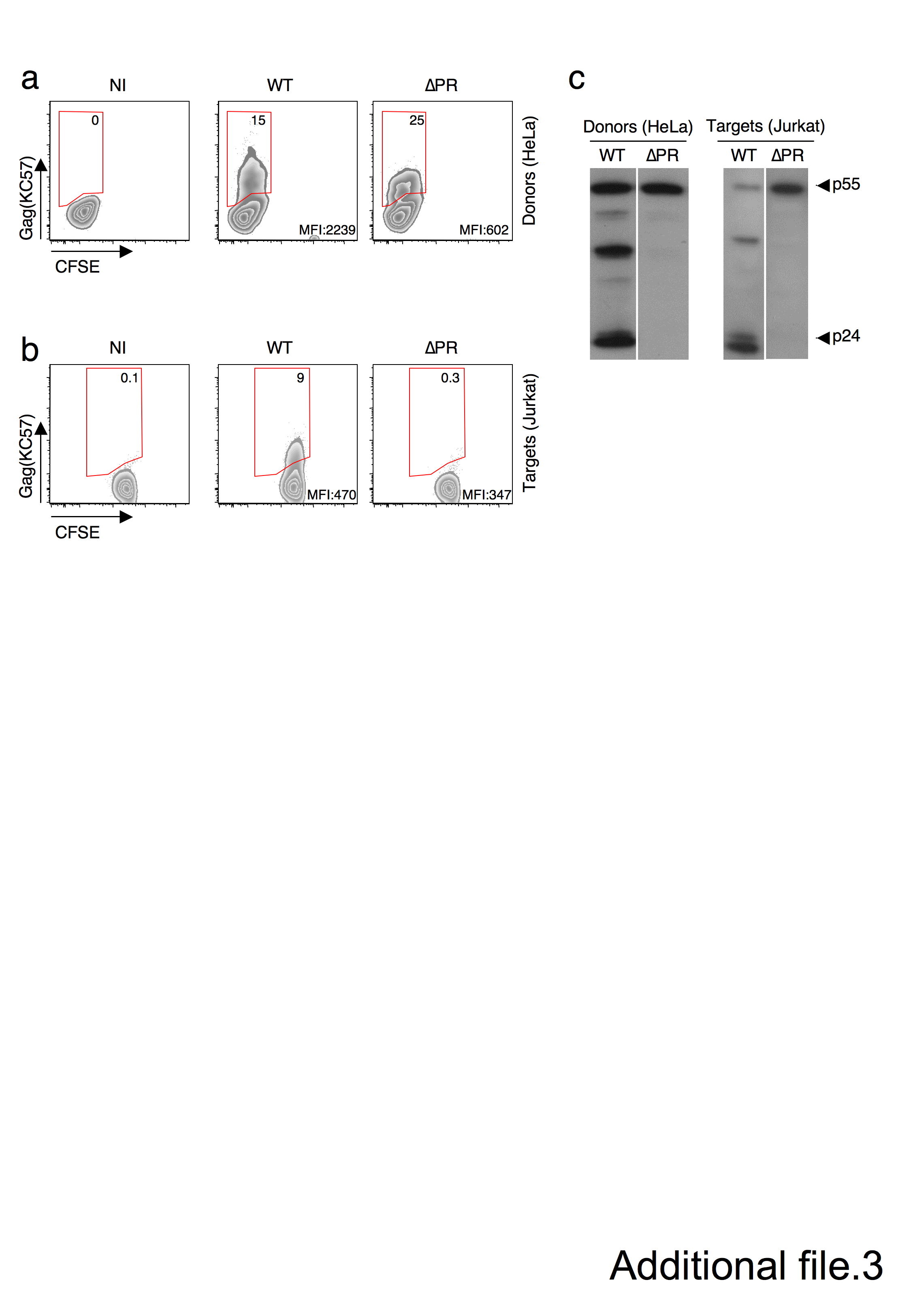

Supplement: Additional file 3 — The monoclonal antibody anti HIV-1 p24 KC57 preferentially recognizes mature p24 by flow cytometry. HeLa cells were transfected with proviral DNA coding for WT, or ∆PR, which is defective for the viral protease. 48h after transfection, HeLa cells were used as donors for a 2h co-culture with Jurkat target cells. (a) Donors were analyzed by flow cytometry using the anti HIV-1 p24 monoclonal antibody KC57. This antibody recognizes both the Gag precursor and mature proteins in donor cells, which over-express the viral proteins. Note that the MFI of the ∆PR provirus is reduced. (b) Targets were analyzed by flow cytometry using the anti HIV-1 p24 KC57 antibody. In target cells the Gag (KC57) signal is visible with the WT virus, and barely detected with ∆PR. (c) Donor and target cells were also harvested separately and analyzed by western blotting using the anti-p24 monoclonal antibody 25A. In donor cells, Gag species from both WT and ∆PR were detected. As expected, ∆PR produced only the Gag precursor (p55). A similar profile of staining was obtained in target Jurkat cells. Of note, KC57, when used in the western blot experiment, also detects both p55 and p24 (not shown). One out of two experiments is shown. [file 1742-4690-10-80-S3.png]
